# Supplementary figures and images for: Heterogeneity in response to serological exposure markers of recent Plasmodium vivax infections in contrasting epidemiological contexts
Source: PLoS Negl Trop Dis. 2021 Feb 16;15(2):e0009165. doi: 10.1371/journal.pntd.0009165 (PMC7909627; doi:10.1371/journal.pntd.0009165)

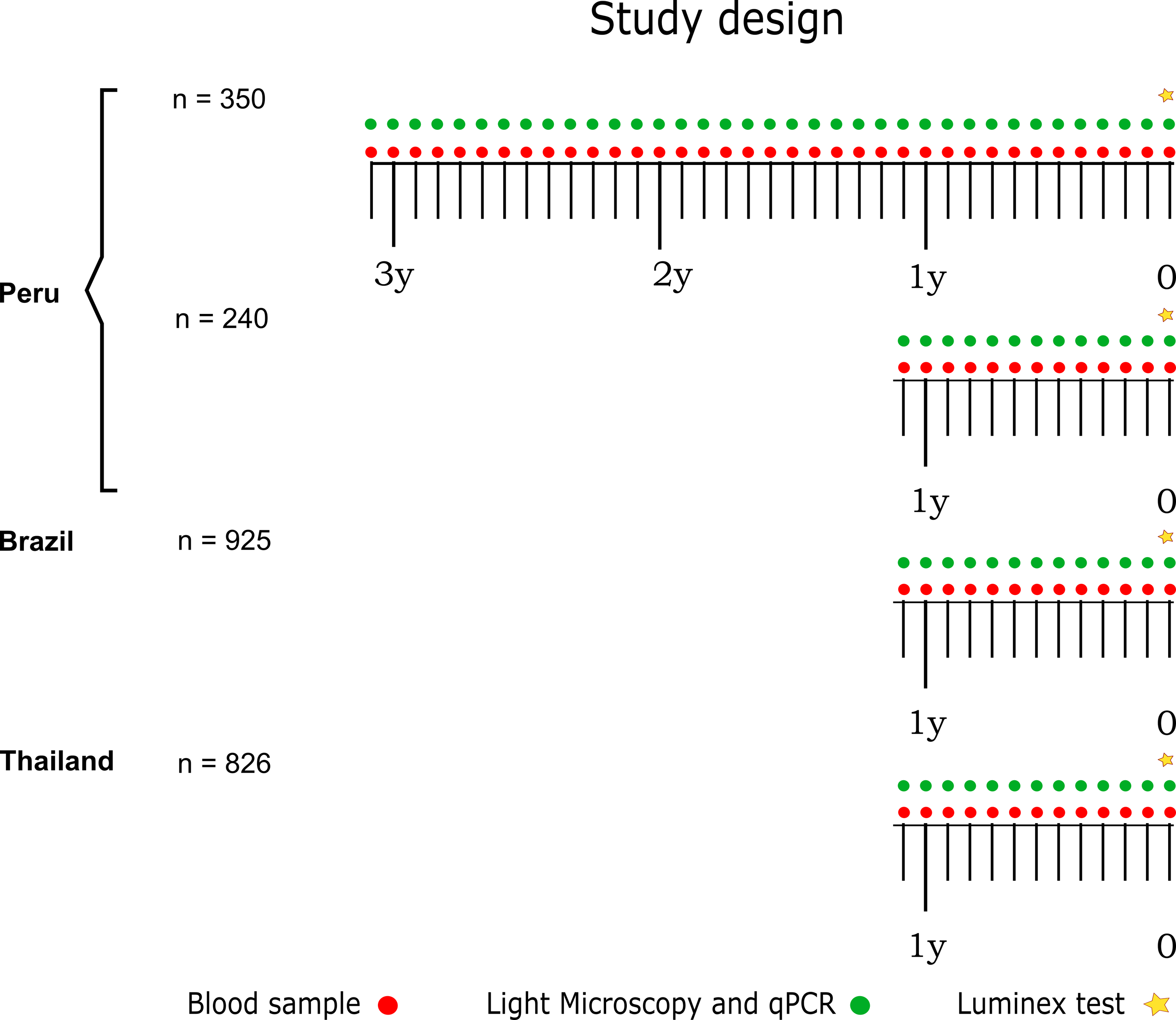

Supplement: S1 Fig — Five hundred ninety (590) individuals from the Peruvian cohort were included from which 350 were followed up to 37 months, and 240 individuals were followed for the last 13 months of the study. For the Brazilian (13 months) and Thai (14 months) cohort, 925 and 826 individuals were included, respectively. In the three cohorts, a monthly blood sample was taken for diagnosis by light microscopy and qPCR. “0” denotes the last time point of following up where a serum sample was taken for Luminex assay. (TIF) [file pntd.0009165.s001.tif]

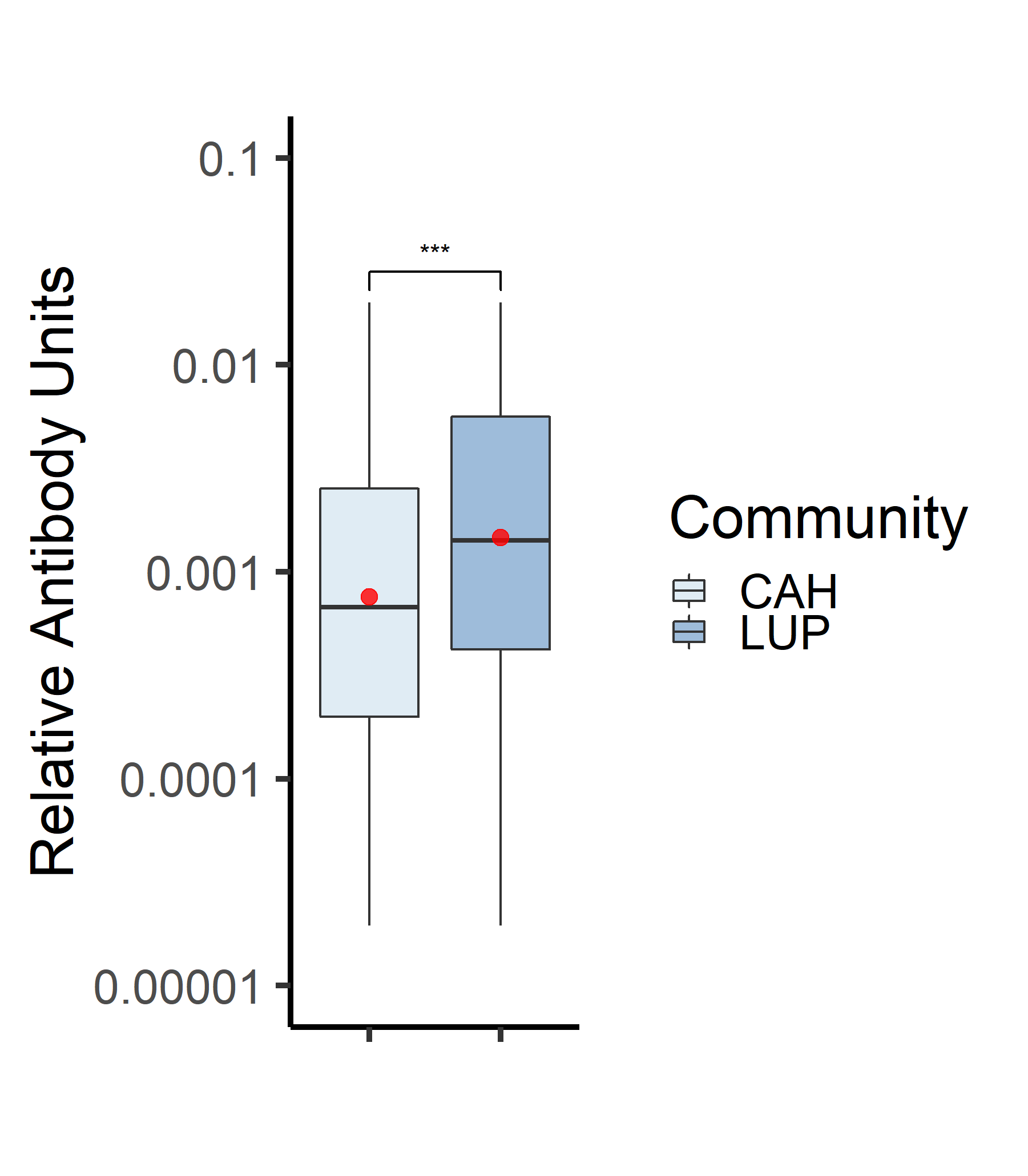

Supplement: S2 Fig — Red dot indicates geometric mean titer (GMT) in each group. *** = significant difference between GMT of CAH and LUP, p < 0.001, t-test. (TIF) [file pntd.0009165.s002.tif]

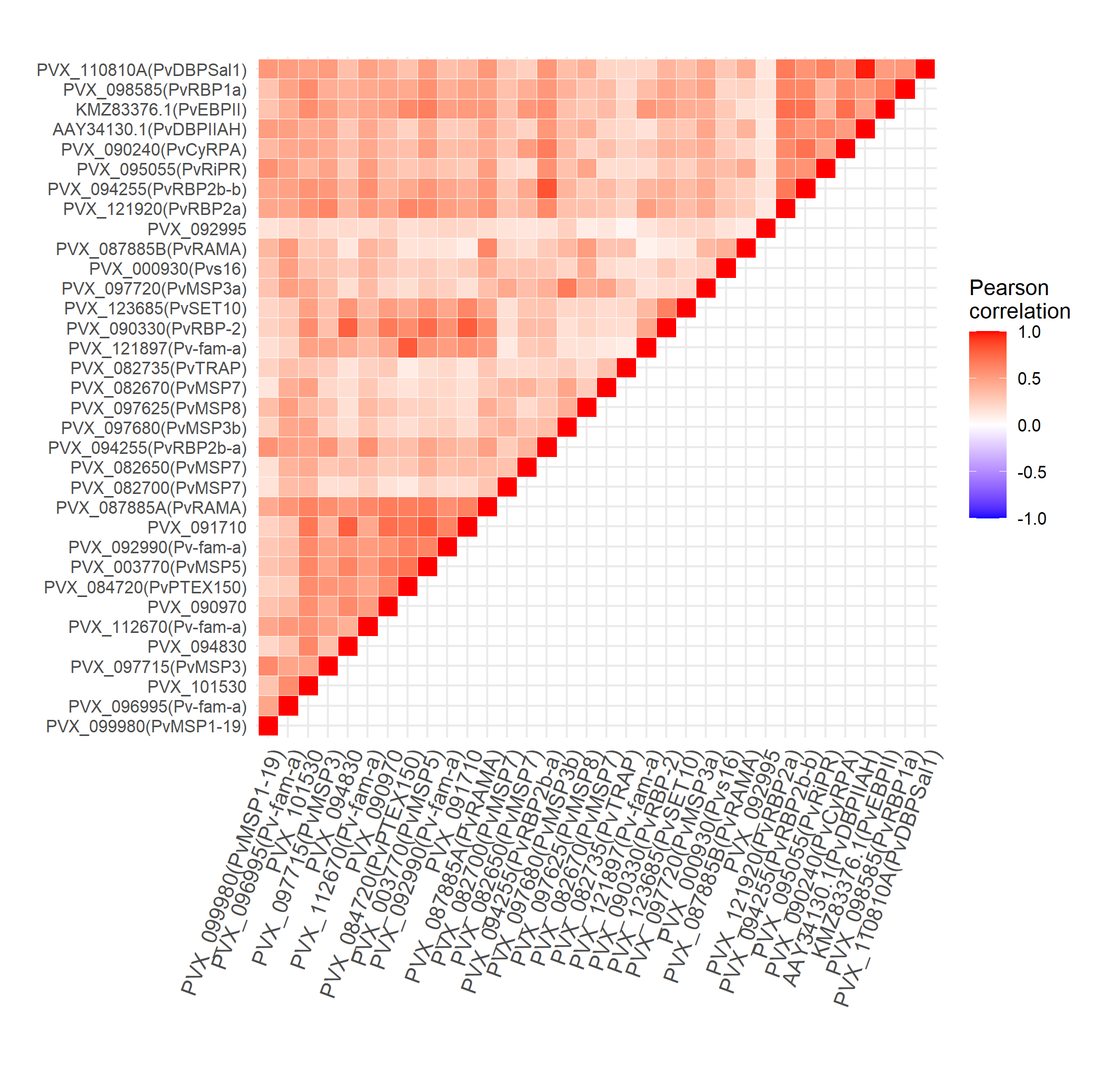

Supplement: S3 Fig — (TIF) [file pntd.0009165.s003.tif]

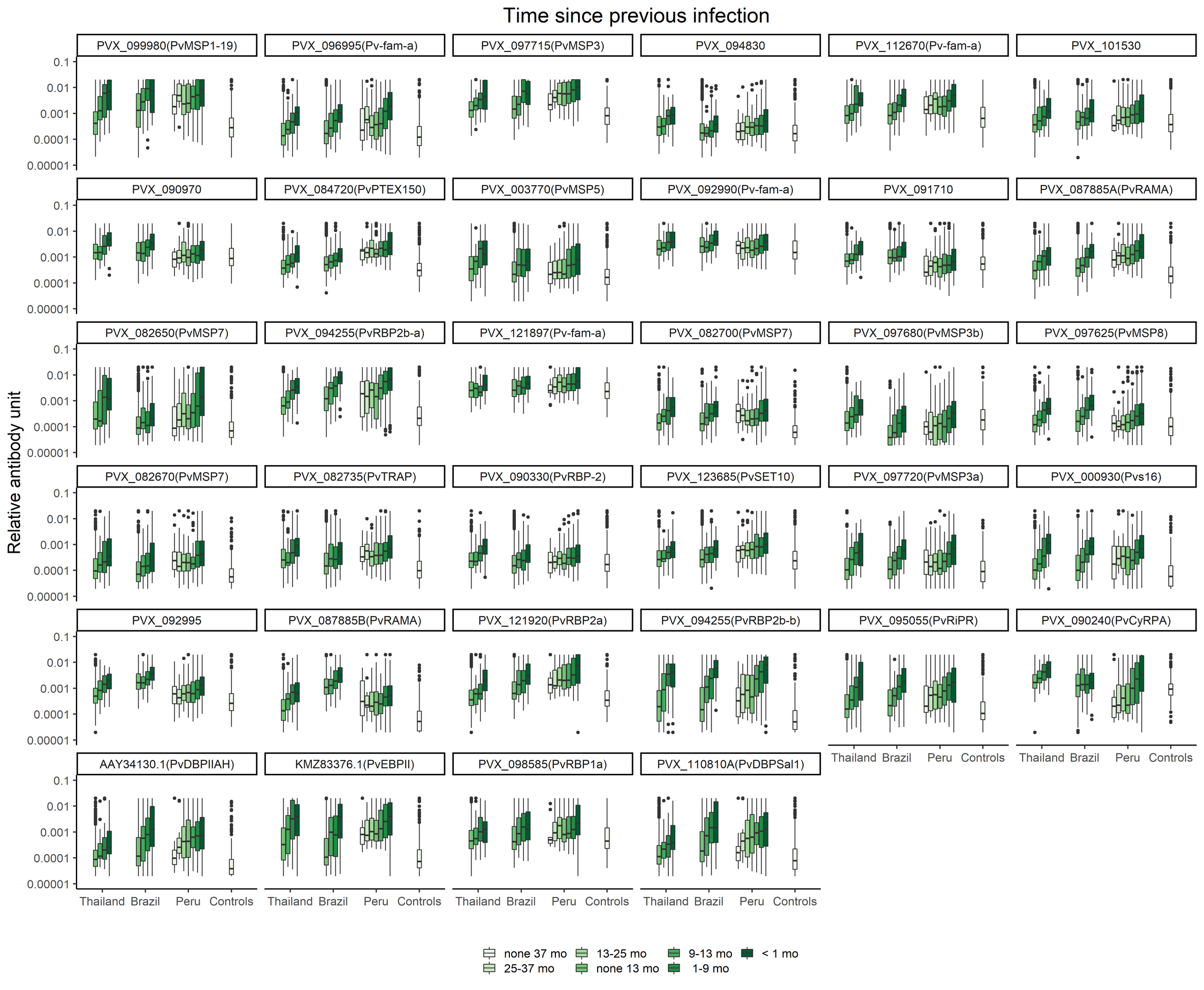

Supplement: S4 Fig — Mo: number of months since previous infection. (TIF) [file pntd.0009165.s004.tif]

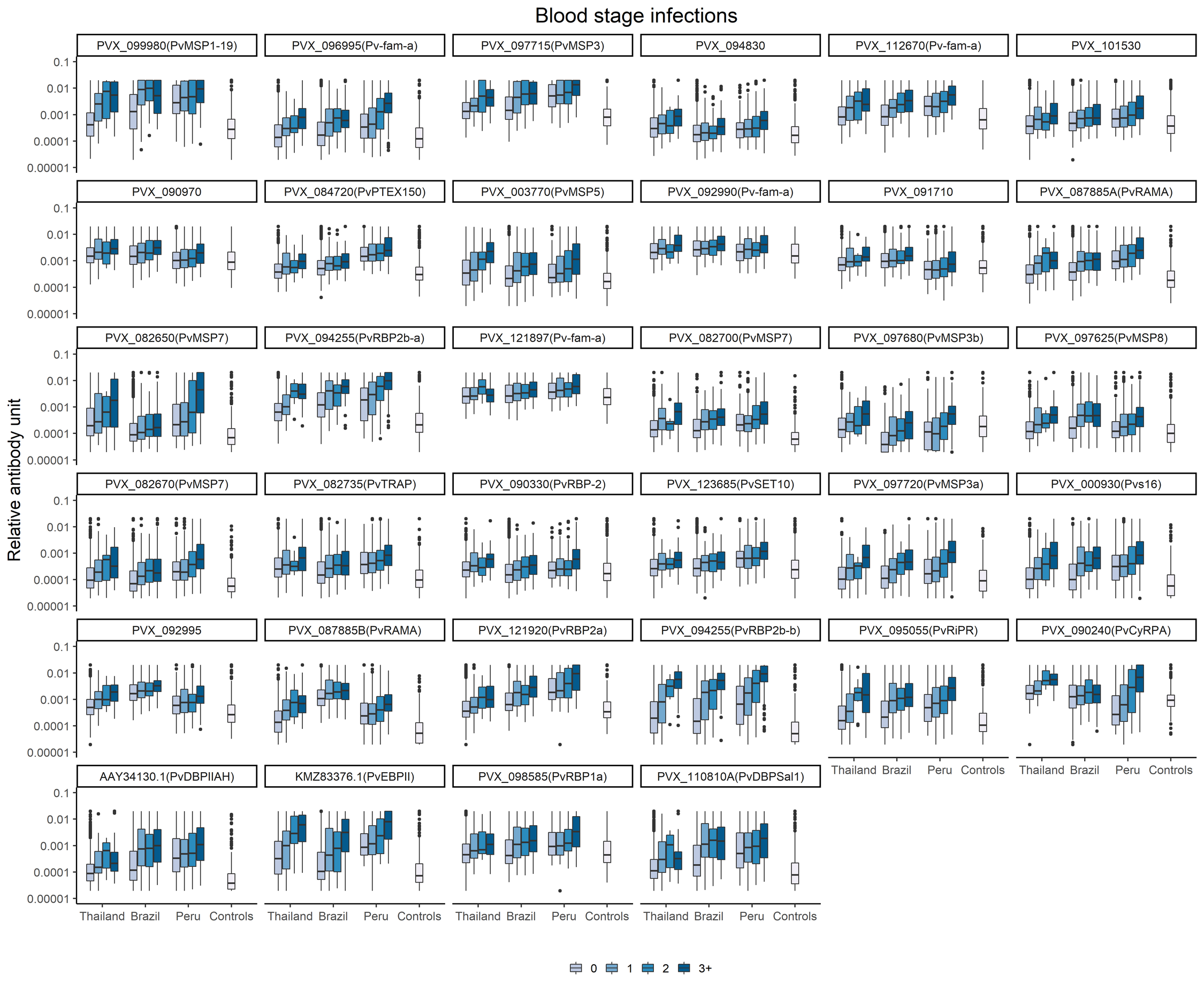

Supplement: S5 Fig — (TIF) [file pntd.0009165.s005.tif]

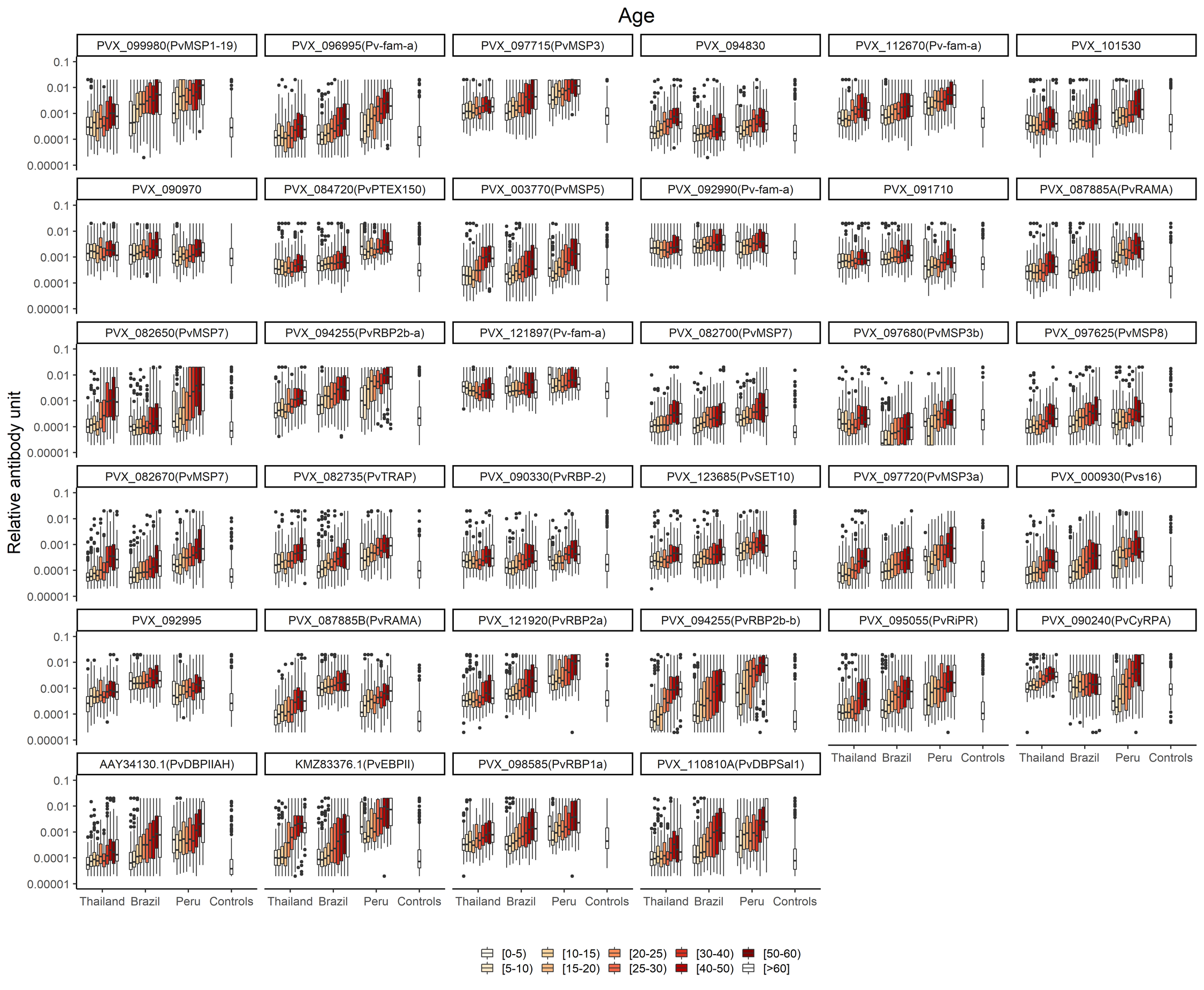

Supplement: S6 Fig — Note the controls are grouped together regardless of age. (TIF) [file pntd.0009165.s006.tif]

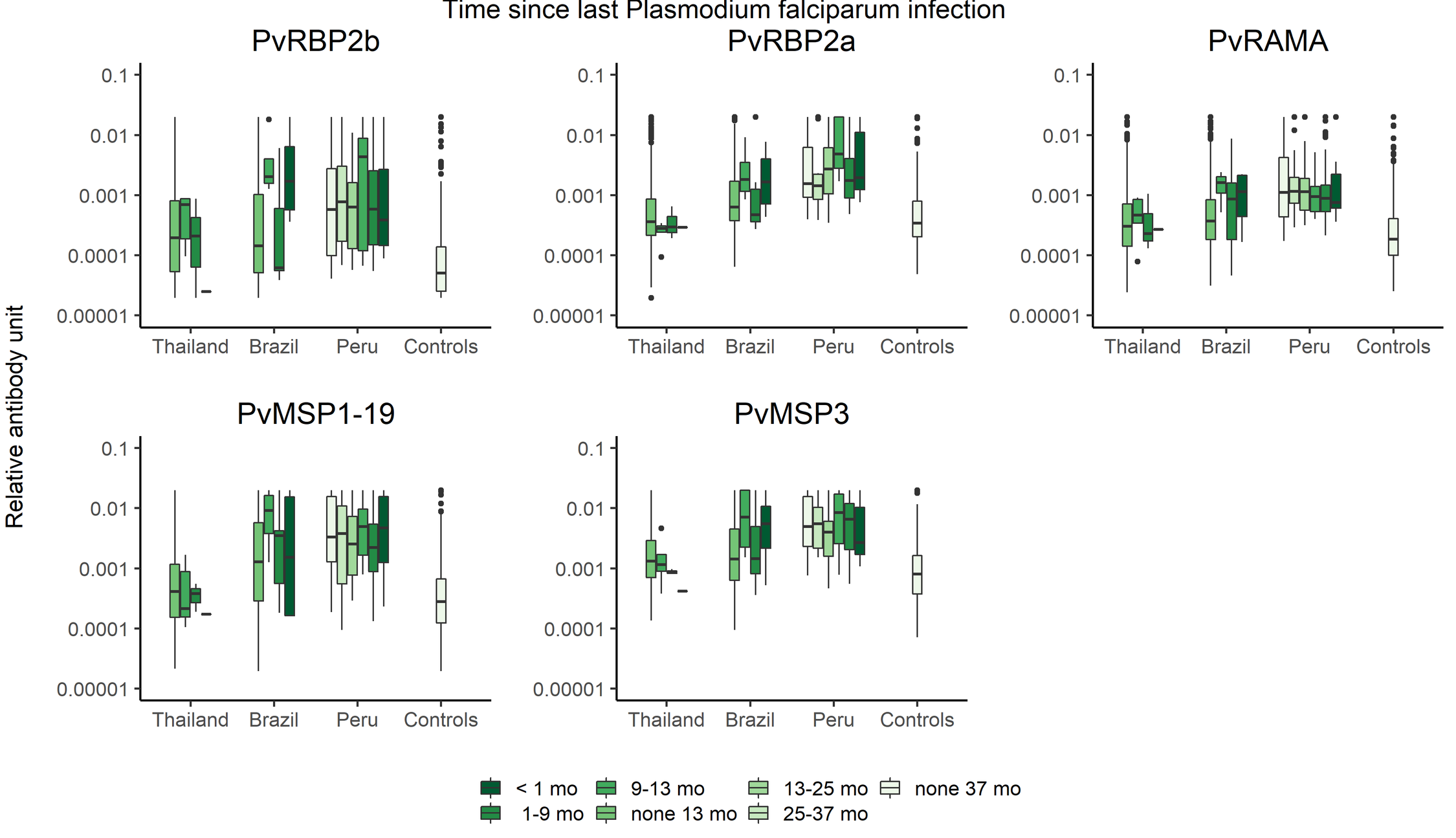

Supplement: S7 Fig — Antibodies responses to PvRBP2b (PVX_094255), PvRBP2 (PVX_121920), PvRAMA (PVX_087885), PvMSP119 (PVX_099980) and PvMSP3 (PVX_097715, hypothetical PvMSP3). Note that all individuals with at least one P. vivax infection were removed from the analysis. There were not significant differences between individuals with P. falciparum PCR detection in the last 9 months and people with no P. falciparum infection. (TIF) [file pntd.0009165.s007.tif]

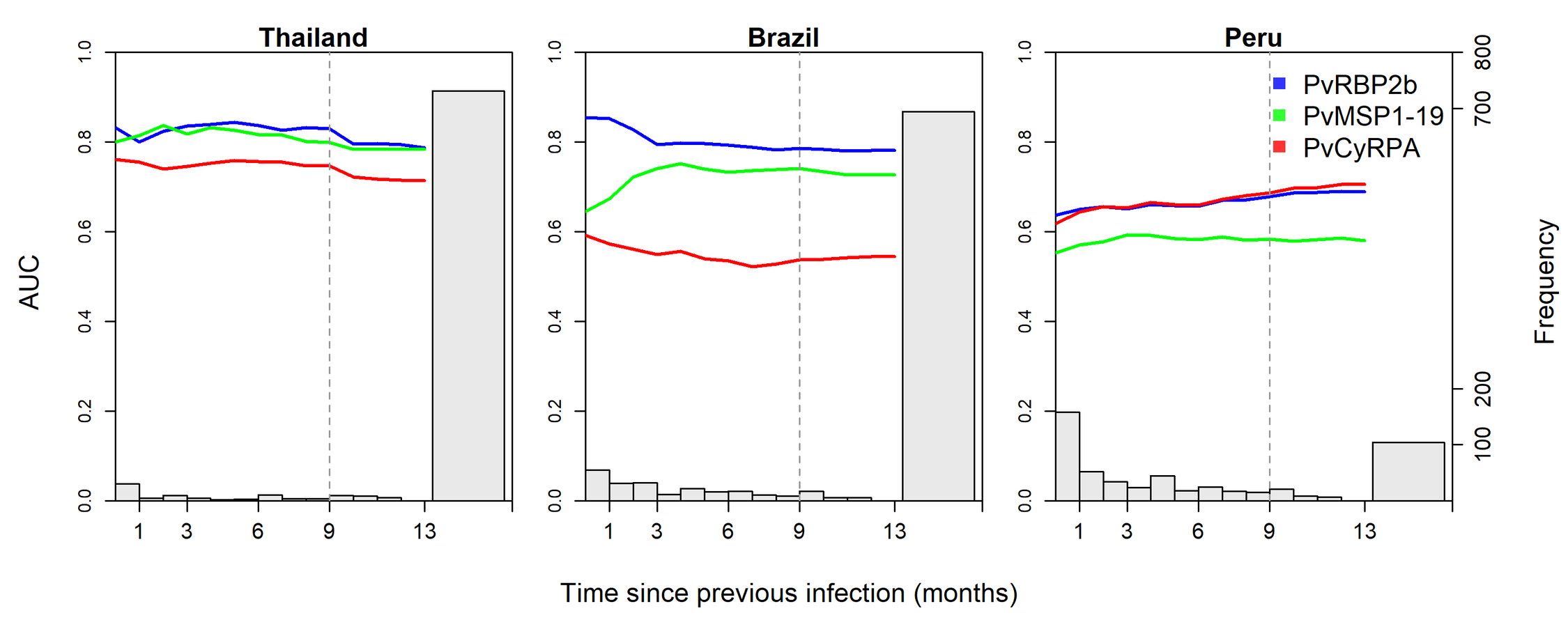

Supplement: S8 Fig — Area under the ROC curve (AUC) values were calculated using data of single antibody responses to PVX_094255 (PvRBP2b), PVX_090240 (PvCyRPA) and PVX_099980 (PvMPS119) to detect infections in each timeframe. Grey bars indicate the frequency of time since previous P. vivax infection in each study site. (TIF) [file pntd.0009165.s008.tif]

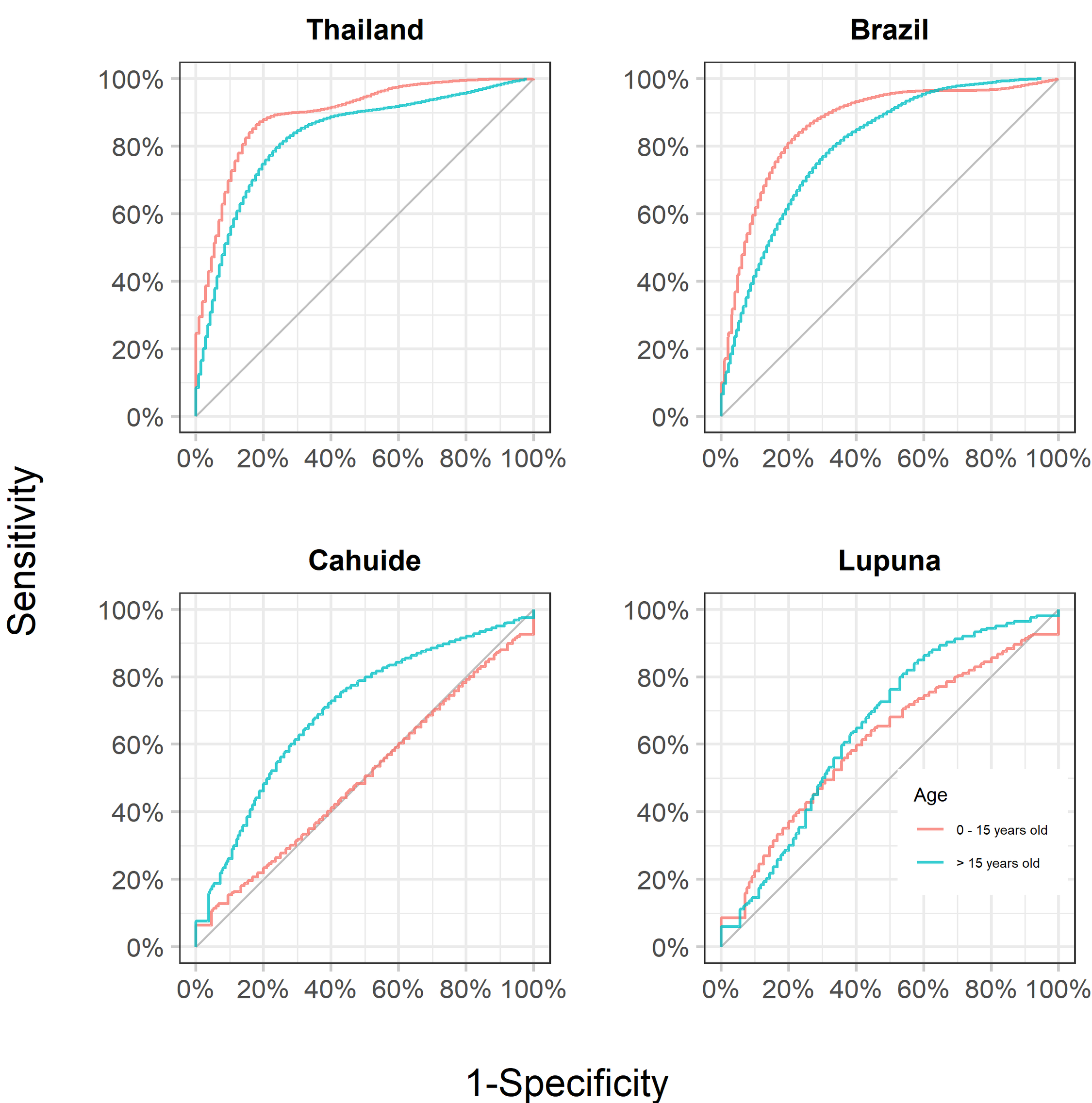

Supplement: S9 Fig — ROC curves displaying the diagnostic performance to detect recent infections given by combinations of the top 5 antibody responses in two age groups: 0–15 years old and >15 years old. Peru cohort data was analyzed according to the Cahuide and Lupuna communities. The turquoise curve represents the ROC curve in individuals older than 15 years old. The red curve represents the ROC curve in individuals younger than 15 years old. Thailand: 0–15 AUC: 0.90, 15+ AUC: 0.83; Brazil 0–15 AUC: 0.87, 15+ AUC: 0.81; Cahuide: 0–15 AUC: 0.51, 15+ AUC: 0.71; Lupuna: 0–15 AUC: 0.62, 15+ AUC: 0.66. (TIF) [file pntd.0009165.s009.tif]
