# Supplementary material for: Heterogeneity in response to serological exposure markers of recent Plasmodium vivax infections in contrasting epidemiological contexts
Source: PLoS Negl Trop Dis. 2021 Feb 16;15(2):e0009165. doi: 10.1371/journal.pntd.0009165 (PMC7909627; doi:10.1371/journal.pntd.0009165)
Supplement: S1 Table — (DOCX) [file pntd.0009165.s010.docx]

| **Table S1. Association of antibody responses with current *P. vivax* infection in the Peruvian cohort.** | | | |
| --- | --- | --- | --- |
| **Antibody response** | **Odds ratio, 95% CI** |  |  |
|  |  |  |  |
| PVX_099980 | 1.27 (0.93,1.74)¥ |  |  |
| PVX_096995 | 1.64 (1.26,2.13)*** |  |  |
| PVX_101530 | 1.43 (1.04,1.98)* |  |  |
| PVX_097715 | 1.59 (1.02,2.49)* |  |  |
| PVX_094830 | 1.55 (1.08,2.22)* |  |  |
| PVX_112670 | 1.99 (1.35,2.92)*** |  |  |
| PVX_090970 | 1.75 (1.18,2.59)** |  |  |
| PVX_084720 | 1.62 (1.07,2.43)* |  |  |
| PVX_003770 | 1.41 (1.06,1.88)* |  |  |
| PVX_092990 | 1.73 (1.11,2.69)* |  |  |
| PVX_091710 | 1.42 (1,2.01)¥ |  |  |
| PVX_087885 | 1.59 (1.11,2.27)* |  |  |
| PVX_082700 | 1.27 (0.91,1.76)¥ |  |  |
| PVX_082650 | 1.38 (1.12,1.7)** |  |  |
| PVX_094255 | 1.62 (1.2,2.18)** |  |  |
| PVX_097680 | 1.51 (1.16,1.96)** |  |  |
| PVX_097625 | 1.34 (1,1.79)¥ |  |  |
| PVX_082670 | 1.29 (0.98,1.71)¥ |  |  |
| PVX_082735 | 1.99 (1.42,2.79)*** |  |  |
| PVX_121897 | 1.71 (1.04,2.81)* |  |  |
| PVX_090330 | 1.50 (1.02,2.19)* |  |  |
| PVX_123685 | 1.48 (1.04,2.1)* |  |  |
| PVX_097720 | 1.60 (1.22,2.08)** |  |  |
| PVX_000930 | 1.49 (1.13,1.96)** |  |  |
| PVX_000930 | 1.82 (1.28,2.59)** |  |  |
| PVX_092995 | 1.24 (0.9,1.7)¥ |  |  |
| PVX_121920 | 1.71 (1.19,2.46)** |  |  |
| PVX_094255B | 1.79 (1.37,2.35)*** |  |  |
| PVX_095055 | 1.46 (1.12,1.89)** |  |  |
| PVX_090240 | 1.64 (1.29,2.09)*** |  |  |
| AAY34130.1 | 1.17 (0.92,1.5)¥ |  |  |
| KMZ83376.1 | 1.43 (1.06,1.93)* |  |  |
| PVX_098585 | 1.56 (1.14,2.14)** |  |  |
| PVX_110810A | 1.19 (0.93,1.53)¥ |  |  |
| 95% CI = 95% confidence interval. ^¥^ : Not significant. *: p < 0.05,**: p < 0.01,***: p < 0.001. Logistic regression models were adjusted by the following covariates: log_10_ (age), gender and working as a farmer. | | | |
