# Supplementary material for: Heterogeneity in response to serological exposure markers of recent Plasmodium vivax infections in contrasting epidemiological contexts
Source: PLoS Negl Trop Dis. 2021 Feb 16;15(2):e0009165. doi: 10.1371/journal.pntd.0009165 (PMC7909627; doi:10.1371/journal.pntd.0009165)
Supplement: S2 Table — (DOCX) [file pntd.0009165.s011.docx]

| **Table S2. Multivariate linear regression model explaining the antibody level in the Peruvian cohort.** The model was corrected by age, community, gender, *P. vivax* infections in the last 6 months, *P. vivax* infections in the initial 7 months, clinical infections in the last 6 months, education level, time living in the community and working as a farmer. | | | | | | | | | | |
| --- | --- | --- | --- | --- | --- | --- | --- | --- | --- | --- |
|  | Age, Log_10_ | Community (Ref = Cahuide) | Gender (Ref = Female) | Number of qPCR +ve last 6 months^¥^ | Number of qPCR +ve initial 7 months^¶^ | Number of clinical *P. vivax* episodes last 6 months | Education (Ref = No education) | | Time in community (years) | Working as farmer |
|  |  |  |  |  |  |  | Primary school | Secondary school or higher |  |  |
|  | Coefficient | Coefficient | Coefficient | Coefficient | Coefficient | Coefficient | Coefficient | Coefficient | Coefficient | Coefficient |
|  | 95% CI | 95% CI | 95% CI | 95% CI | 95% CI | 95% CI | 95% CI | 95% CI | 95% CI | 95% CI |
| PVX_099980 | 0.420*** |  |  | 0.094*** |  | 0.416** |  |  |  |  |
|  | 0.267, 0.572 |  |  | 0.055, 0.134 |  | 0.178, 0.654 |  |  |  |  |
| PVX_096995 | 0.861*** | 0.305*** |  | 0.167*** |  | 0.384** |  |  |  |  |
|  | 0.705, 1.017 | 0.197, 0.414 |  | 0.126, 0.207 |  | 0.129, 0.640 |  |  |  |  |
| PVX_101530 | 0.242** |  | 0.157** | 0.069*** |  |  |  |  | 0.007** |  |
|  | 0.072, 0.413 |  | 0.066, 0.247 | 0.032, 0.106 |  |  |  |  | 0.003, 0.010 |  |
| PVX_097715 | 0.336*** | 0.096** |  | 0.061*** |  |  |  |  |  |  |
|  | 0.233, 0.439 | 0.024, 0.168 |  | 0.031, 0.090 |  |  |  |  |  |  |
| PVX_094830 | 0.374*** | 0.175*** | 0.122** | 0.060** |  |  |  |  |  |  |
|  | 0.251, 0.496 | 0.094, 0.255 | 0.040, 0.203 | 0.023, 0.098 |  |  |  |  |  |  |
| PVX_112670 | 0.396*** |  | 0.105* | 0.109*** |  |  |  |  |  |  |
|  | 0.277, 0.515 |  | 0.023, 0.186 | 0.076, 0.142 |  |  |  |  |  |  |
| PVX_090970 | 0.280*** |  | 0.112** | 0.056** |  |  |  |  |  |  |
|  | 0.161, 0.398 |  | 0.033, 0.191 | 0.023, 0.088 |  |  |  |  |  |  |
| PVX_084720 | 0.242*** |  | 0.144*** |  | 0.040** |  |  |  |  |  |
|  | 0.136, 0.349 |  | 0.069, 0.219 |  | 0.010, 0.070 |  |  |  |  |  |
| PVX_003770 | 0.705*** | 0.219*** |  |  | 0.073** |  |  |  |  |  |
|  | 0.556, 0.853 | 0.113, 0.324 |  |  | 0.031, 0.115 |  |  |  |  |  |
| PVX_092990 | 0.257*** |  |  | 0.062*** |  |  |  |  |  |  |
|  | 0.150, 0.364 |  |  | 0.033, 0.092 |  |  |  |  |  |  |
| PVX_091710 |  | 0.175*** | 0.136** |  |  |  | 0.115● | 0.189** |  | 0.193** |
|  |  | 0.089, 0 .262 | 0.049, 0.223 |  |  |  | -0.003, 0.232 | 0.059, 0.320 |  | 0.074, 0.312 |
| PVX_087885 | 0.320*** | 0.125** | 0.145** | 0.074*** |  |  |  |  |  |  |
|  | 0.197, 0.443 | 0.041, 0.210 | 0.062, 0.228 | 0.038, 0.109 |  |  |  |  |  |  |
| PVX_082700 | 0.514*** | 0.200*** |  |  | 0.068** |  |  |  |  |  |
|  | 0.385, 0.642 | 0.107, 0.293 |  |  | 0.024, 0.111 |  |  |  |  |  |
| PVX_082650 | 1.127*** | 0.538*** | 0.177** | 0.106** | 0.118*** |  |  |  |  |  |
|  | 0.950, 1.305 | 0.411, 0.666 | 0.060, 0.294 | 0.046, 0.166 | 0.068, 0.167 |  |  |  |  |  |
| PVX_094255A | 0.709*** | 0.400*** |  | 0.138*** |  | 0.505*** |  |  |  |  |
|  | 0.556, 0.861 | 0.297, 0.502 |  | 0.100, 0.177 |  | 0.284, 0.727 |  |  |  |  |
| PVX_097680 | 0.659*** | 0.234*** | 0.202*** | 0.177*** |  |  |  |  |  |  |
|  | 0.500, 0.818 | 0.126, 0.342 | 0.096, 0.307 | 0.130, 0.224 |  |  |  |  |  |  |
| PVX_097625 |  |  |  | 0.099*** |  |  |  |  | 0.009*** |  |
|  |  |  |  | 0.056, 0.143 |  |  |  |  | 0.005, 0.012 |  |
| PVX_082670 | 0.515*** | 0.204*** | 0.156** | 0.095*** |  |  |  |  | 0.006** |  |
|  | 0.337, 0.694 | 0.091, 0.316 | 0.057, 0.255 | 0.051, 0.139 |  |  |  |  | 0.001, 0.010 |  |
| PVX_082735 | 0.505*** |  |  | 0.106*** |  |  |  |  |  |  |
|  | 0.377, 0.633 |  |  | 0.064, 0.149 |  |  |  |  |  |  |
| PVX_121897 | 0.146** |  | 0.130*** | 0.042** |  |  |  |  |  |  |
|  | 0.056, 0.236 |  | 0.066, 0.193 | 0.015,0.069 |  |  |  |  |  |  |
| PVX_090330 | 0.403*** | 0.145*** | 0.143*** |  | 0.059** |  |  |  |  |  |
|  | 0.290, 0.517 | 0.064, 0.226 | 0.067, 0.219 |  | 0.024, 0.092 |  |  |  |  |  |
| PVX_123685 | 0.291*** | 0.135** | 0.126** |  | 0.050** |  |  |  |  |  |
|  | 0.164, 0.417 | 0.044, 0.227 | 0.039, 0.212 |  | 0.015, 0.085 |  |  |  |  |  |
| PVX_097720 | 0.756*** | 0.226*** |  | 0.159*** | 0.063** |  |  |  |  |  |
|  | 0.600, 0.912 | 0.116, 0.335 |  | 0.114, 0.204 | 0.020, 0.107 |  |  |  |  |  |
| PVX_000930 | 0.515*** | 0.247*** |  | 0.112*** |  |  |  |  |  |  |
|  | 0.361, 0.670 | 0.138, 0.356 |  | 0.068, 0.157 |  |  |  |  |  |  |
| PVX_095055 | 0.524*** | 0.214*** |  | 0.133*** |  | 0.504** |  |  |  |  |
|  | 0.355, 0.693 | 0.095, 0.332 |  | 0.082, 0.185 |  | 0.187, 0.821 |  |  |  |  |
| PVX_090240 | 1.062*** | 0.466*** |  | 0.174*** | 0.073** |  |  |  |  |  |
|  | 0.901, 1.222 | 0.352, 0.581 |  | 0.127, 0.221 | 0.028, 0.118 |  |  |  |  |  |
| PVX_110810A | 0.627*** |  |  | 0.083** |  |  |  |  |  |  |
|  | 0.449, 0.805 |  |  | 0.029, 0.136 |  |  |  |  |  |  |
| AAY34130.1 | 0.667*** |  |  | 0.080** |  |  |  |  |  |  |
|  | 0.491, 0.843 |  |  | 0.027, 0.134 |  |  |  |  |  |  |
| KMZ83376.1 | 0.761*** | 0.268*** |  | 0.071*** | 0.078*** |  |  |  |  |  |
|  | 0.624, 0.897 | 0.172, 0.363 |  | 0.031, 0.111 | 0.038, 0.118 |  |  |  |  |  |
| PVX_098585 | 0.613*** | 0.129*** | 0.169*** | 0.072** | 0.055** |  |  |  |  |  |
|  | 0.482, 0.745 | 0.034, 0.223 | 0.080, 0.259 | 0.029, 0.115 | 0.014, 0.095 |  |  |  |  |  |
| PVX_121920 | 0.512*** |  |  | 0.094*** |  |  |  |  |  |  |
|  | 0.390, 0.633 |  |  | 0.058, 0.129 |  |  |  |  |  |  |
| PVX_094255B | 0.917*** | 0.502*** |  | 0.194*** |  | 0.449*** |  |  |  |  |
|  | 0.750, 1.083 | 0.388, 0.616 |  | 0.154, 0.234 |  | 0.204, 0.693 |  |  |  |  |
| PVX_087885 | 0.446*** | 0.201*** |  | 0.074*** |  | 0.419** |  |  |  |  |
|  | 0.309, 0.583 | 0.105, 0.297 |  | 0.037, 0.111 |  | 0.159, 0.679 |  |  |  |  |
| PVX_092995 | 0.264*** |  | 0.173*** | 0.093*** |  |  |  |  |  |  |
|  | 0.135, 0.393 |  | 0.086, 0.260 | 0.060, 0.126 |  |  |  |  |  |  |
| A clinical episode was defined by having a temperature of ≥38.0°C at the time of sample collection or having fever in the past 48 hours and parasite density >500 parasites/uL. ^¥^: Number of *P. vivax* positive qPCR results within the last six months of following up. Concurrent *P. vivax* infections were included in the variable Number of *P. vivax* positive qPCR results within the last six months of following up. ^¶^ : Number of *P. vivax* positive qPCR results in the initial seven months of following up. 95% CI: 95% confidence interval. * p<0.05; **p<0.01; ***p<0.001. | | | | | | | | | | |
|  |  |  |  |  |  |  |  |  |  |  |
