# Supplementary material for: Heterogeneity in response to serological exposure markers of recent Plasmodium vivax infections in contrasting epidemiological contexts
Source: PLoS Negl Trop Dis. 2021 Feb 16;15(2):e0009165. doi: 10.1371/journal.pntd.0009165 (PMC7909627; doi:10.1371/journal.pntd.0009165)
Supplement: S3 Table — (DOCX) [file pntd.0009165.s012.docx]

| **Table S3. Epidemiologic characteristics of the study sites and participants.** | |  |  |
| --- | --- | --- | --- |
| Characteristics | Thailand n = 826 | Brazil n = 925 | Peru n = 590 |
| Age (median, range) | 24.0 (1.00 - 78.0) | 25.37 (0.01 - 102.64) | 26.0 ( 3.80 - 85.57) |
| <15 years old (%) | 40.68 | 37.84 | 35.93 |
| 15-39 years old (%) | 32.32 | 30.05 | 32.54 |
| ≥40 years old (%) | 27.00 | 32.11 | 31.53 |
| Female sex (%) | 54.6 | 50.59 | 58.30 |
| Forest-related occupation (≥18 years old) (%)^¥^ |  |  |  |
| Farmer/Fishing ^a,c^ | 33.17 | 14.55 | 38.32 |
| Infection by qPCR at D0^ⱡ^ |  |  |  |
| *P. vivax* (%) ^a, b^ | 3.03 | 4.22 | 21.19 |
| *P. falciparum* (%) ^a, b^ | 0.24 | 0.43 | 2.71 |
| Months of follow-up, *n* | 14 | 13 | 13/37^#^ |
| Total clinical *P. vivax* infections, *n* (%) |  |  |  |
| 0 ^c,d^ | 801 (96.97) | 846 (91.46) | 562 (95.25) |
| 1 ^c^ | 22 (2.66) | 56 (6.05) | 26 (4.41) |
| ≥2 ^c,d^ | 3 (0.36) | 23 (2.49) | 2 (0.34) |
| Total *P. vivax* infections by qPCR in 13 months, *n* (%) |  |  |  |
| 0 ^a, b^ | 729 (88.26) | 690 (74.59) | 146(24.74) |
| 1 ^a, b^ | 41 (4.96) | 98 (10.60) | 170 (28.81) |
| 2 ^a, b^ | 10 (1.21) | 54 (5.84) | 117 (19.83) |
| ≥3 ^a, b^ | 46 (5.57) | 83 (8.97) | 157 (26.61) |
| Days since last past *P. vivax* infection, *n* (%) |  |  |  |
| < 1 month ^a, b^ | 30 (3.63) | 55 (5.95) | 160 (27.12) |
| 1-9 months ^a, b^ | 42 (5.08) | 149 (16.11) | 228 (38.64) |
| 9-13 months ^a, b^ | 25 (3.03) | 31 (3.35) | 56 (9.49) |
| No infection in 13 months ^a, b^ | 729 (88.26) | 690 (74.59) | 146 (24.74) |
| ^¥^ Occupation in Peru: Farmer (30.83%), fishing (1.73%), woodcutter (5.76%). ^#^: Individuals followed for 13 months (n=240), individuals followed for 37 months (n=350). ^a^: Significant differences between Thailand and Peru, p<0.05; ^b^: Significant differences between Brazil and Peru, p<0.05; ^c^: Significant differences between Thailand and Brazil, p<0.05; ^d^: Significant differences between Brazil and Peru, p<0.05. ^ⱡ^ Prevalence includes single - and mixed-species infections. | | | |
|  |  |  |  |
|  |  |  |  |
