# Supplementary material for: Heterogeneity in response to serological exposure markers of recent Plasmodium vivax infections in contrasting epidemiological contexts
Source: PLoS Negl Trop Dis. 2021 Feb 16;15(2):e0009165. doi: 10.1371/journal.pntd.0009165 (PMC7909627; doi:10.1371/journal.pntd.0009165)
Supplement: S4 Table — (DOCX) [file pntd.0009165.s013.docx]

| **Table S4. Characteristic of evaluated constructs.** | |  |  |  |  |  |  |
| --- | --- | --- | --- | --- | --- | --- | --- |
| Protein ID^a^ | Gene Annotation^a^ | AUC | | | | | Rank |
|  |  | Thailand | Brazil | Peru | Average | Combined data set |  |
| PVX_094255B | reticulocyte binding protein 2b (RBP2b) | 0.83 | 0.79 | 0.68 | 0.77 | 0.80 | 1 |
| PVX_121920 | reticulocyte binding protein 2a (RBP2a) | 0.69 | 0.70 | 0.61 | 0.67 | 0.77 | 2 |
| PVX_087885B | rhoptry-associated membrane antigen, putative | 0.74 | 0.72 | 0.61 | 0.69 | 0.76 | 3 |
| PVX_099980 | merozoite surface protein 1 (MSP1-19) | 0.80 | 0.74 | 0.58 | 0.71 | 0.76 | 4 |
| PVX_097715 | hypothetical protein | 0.72 | 0.73 | 0.59 | 0.68 | 0.76 | 5 |
| KMZ83376.1^b^ | erythrocyte binding protein II (PvEBPII) | 0.76 | 0.72 | 0.62 | 0.70 | 0.75 | 6 |
| PVX_000930 | sexual stage antigen s16, putative | 0.77 | 0.75 | 0.63 | 0.72 | 0.75 | 7 |
| PVX_094255A | reticulocyte binding protein 2b (RBP2b) | 0.79 | 0.72 | 0.66 | 0.72 | 0.75 | 8 |
| PVX_096995 | tryptophan-rich antigen (Pv-fam-a) | 0.74 | 0.71 | 0.66 | 0.70 | 0.75 | 9 |
| PVX_084720 | translocon component PTEX150, putative | 0.72 | 0.62 | 0.56 | 0.64 | 0.74 | 10 |
| AAY34130.1b | Duffy binding protein (DBP, region 2, AH strain) | 0.71 | 0.73 | 0.58 | 0.67 | 0.74 | 11 |
| PVX_095055 | Rh5 interacting protein, putative (RIPR) | 0.74 | 0.75 | 0.62 | 0.70 | 0.74 | 12 |
| PVX_112670 | unspecified product | 0.75 | 0.72 | 0.62 | 0.69 | 0.73 | 13 |
| PVX_110810A | Duffy binding protein (DBP, region 2, Sal1 strain) | 0.70 | 0.73 | 0.58 | 0.67 | 0.73 | 14 |
| PVX_097720 | merozoite surface protein 3 (MSP3.10) | 0.78 | 0.71 | 0.65 | 0.72 | 0.72 | 15 |
| PVX_082670 | merozoite surface protein 7 (MSP7), putative | 0.70 | 0.68 | 0.63 | 0.67 | 0.71 | 16 |
| PVX_082700 | merozoite surface protein 7 (MSP7.1) | 0.72 | 0.70 | 0.61 | 0.68 | 0.70 | 17 |
| PVX_098585 | reticulocyte binding protein 1a (RBP1a) | 0.67 | 0.67 | 0.59 | 0.64 | 0.69 | 18 |
| PVX_097625 | merozoite surface protein 8 (MSP8), putative | 0.76 | 0.72 | 0.63 | 0.70 | 0.68 | 19 |
| PVX_123685 | histone-lysine N-methyltransferase, H3 lysine-4 specific (SET10), putative | 0.68 | 0.61 | 0.56 | 0.62 | 0.68 | 20 |
| PVX_097680 | merozoite surface protein 3 (MSP3.3) | 0.72 | 0.72 | 0.63 | 0.69 | 0.66 | 21 |
| PVX_101530 | Plasmodium exported protein, unknown function | 0.69 | 0.59 | 0.58 | 0.62 | 0.65 | 22 |
| PVX_082735 | thrombospondin-related anonymous protein (TRAP) | 0.66 | 0.63 | 0.59 | 0.63 | 0.65 | 23 |
| PVX_082650 | merozoite surface protein 7 (MSP7), putative | 0.69 | 0.59 | 0.64 | 0.64 | 0.65 | 24 |
| PVX_003770 | merozoite surface protein 5 (MSP5) | 0.73 | 0.65 | 0.61 | 0.66 | 0.64 | 25 |
| PVX_121897 | tryptophan-rich antigen (Pv-fam-a) | 0.55 | 0.57 | 0.56 | 0.56 | 0.62 | 26 |
| PVX_087885A | rhoptry associated membrane antigen, putative | 0.78 | 0.66 | 0.61 | 0.68 | 0.62 | 27 |
| PVX_090330 | reticulocyte binding protein 2 precursor (PvRBP-2), putative | 0.71 | 0.61 | 0.58 | 0.63 | 0.62 | 28 |
| PVX_092995 | tryptophan-rich antigen (Pv-fam-a) | 0.77 | 0.62 | 0.60 | 0.67 | 0.61 | 29 |
| PVX_092990 | tryptophan-rich antigen (Pv-fam-a) | 0.66 | 0.59 | 0.60 | 0.61 | 0.59 | 30 |
| PVX_094830 | hypothetical protein, conserved | 0.69 | 0.58 | 0.58 | 0.62 | 0.59 | 31 |
| PVX_090240 | cysteine-rich protective antigen, putative (CyRPA) | 0.75 | 0.54 | 0.69 | 0.66 | 0.58 | 32 |
| PVX_090970 | hypothetical protein, conserved | 0.69 | 0.61 | 0.55 | 0.62 | 0.55 | 33 |
| PVX_091710 | hypothetical protein, conserved | 0.72 | 0.58 | 0.56 | 0.62 | 0.52 | 34 |
|  |  |  |  |  |  |  |  |
| ^aPlasmoDB^ release 36 (http://plasmodb.org/plasmo/), ^bGenBank^ | |  |  |  |  |  |  |
|  |  |  |  |  |  |  |  |
| **References** |  |  |  |  |  |  |  |
| 1 | J. Hietanen, A. Chim-Ong, T. Chiramanewong, J. Gruszczyk, W. Roobsoong, W. H. Tham, J. Sattabongkot, W. Nguitragool, Gene Models, Expression Repertoire, and Immune Response of Plasmodium vivax Reticulocyte Binding Proteins. *Infect Immun* **84,** 677-685 (2015). | | | | | | |
| 2 | F. Lu, J. Li, B. Wang, Y. Cheng, D. H. Kong, L. Cui, K. S. Ha, J. Sattabongkot, T. Tsuboi, E. T. Han, Profiling the humoral immune responses to Plasmodium vivax infection and identification of candidate immunogenic rhoptry-associated membrane antigen (RAMA). *J Proteomics* **102,** 66-82 (2014). | | | | | | |
| 3 | J. Healer, J. K. Thompson, D. T. Riglar, D. W. Wilson, Y. H. Chiu, K. Miura, L. Chen, A. N. Hodder, C. A. Long, D. S. Hansen, J. Baum, A. F. Cowman, Vaccination with conserved regions of erythrocyte-binding antigens induces neutralizing antibodies against multiple strains of Plasmodium falciparum. *PLoS One* **8,** e72504 (2013). | | | | | | |
| 4 | J. L. Cole-Tobian, P. Michon, M. Biasor, J. S. Richards, J. G. Beeson, I. Mueller, C. L. King, Strain-specific duffy binding protein antibodies correlate with protection against infection with homologous compared to heterologous plasmodium vivax strains in Papua New Guinean children. *Infect Immun* **77,** 4009-4017 (2009). | | | | | | |
| 5 | C. T. Franca, M. T. White, W. Q. He, J. B. Hostetler, J. Brewster, G. Frato, I. Malhotra, J. Gruszczyk, C. Huon, E. Lin, B. Kiniboro, A. Yadava, P. Siba, M. R. Galinski, J. Healer, C. Chitnis, A. F. Cowman, E. Takashima, T. Tsuboi, W. H. Tham, R. M. Fairhurst, J. C. Rayner, C. L. King, I. Mueller, Identification of highly-protective combinations of Plasmodium vivax recombinant proteins for vaccine development. *Elife* **6,** (2017). | | | | | | |
