# Supplementary material for: Heterogeneity in response to serological exposure markers of recent Plasmodium vivax infections in contrasting epidemiological contexts
Source: PLoS Negl Trop Dis. 2021 Feb 16;15(2):e0009165. doi: 10.1371/journal.pntd.0009165 (PMC7909627; doi:10.1371/journal.pntd.0009165)
Supplement: S5 Table — (DOCX) [file pntd.0009165.s014.docx]

| **Table S5. Top antibody responses for classifying recent infections.** | | | |
| --- | --- | --- | --- |
| **Country** | **Rank** | **Single antibody response model** | **AUC** |
|  |  |  |  |
| **Thailand** | 1 | PVX_094255B | 0.83 |
|  | 2 | PVX_099980 | 0.80 |
|  | 3 | PVX_094255A | 0.79 |
|  | 4 | PVX_097720 | 0.78 |
|  | 5 | PVX_087885A | 0.78 |
|  | 6 | PVX_000930 | 0.77 |
|  | 7 | PVX_092995 | 0.77 |
|  | 8 | KMZ83376.1 | 0.76 |
|  | 9 | PVX_097625 | 0.76 |
|  |  |  |  |
| **Brazil** | 1 | PVX_094255B | 0.79 |
|  | 2 | PVX_000930 | 0.75 |
|  | 3 | PVX_095055 | 0.75 |
|  | 4 | PVX_099980 | 0.74 |
|  | 5 | PVX_097715 | 0.73 |
|  | 6 | AAY34130.1 | 0.73 |
|  | 7 | PVX_110810A | 0.73 |
|  | 8 | PVX_087885 | 0.72 |
|  |  |  |  |
| **Peru** | 1 | PVX_090240 | 0.69 |
|  | 2 | PVX_094255B | 0.68 |
|  | 3 | PVX_096995 | 0.66 |
|  | 4 | PVX_094255A | 0.66 |
|  | 5 | PVX_097720 | 0.65 |
|  | 6 | PVX_082650 | 0.64 |
|  | 7 | PVX_082670 | 0.63 |
|  | 8 | PVX_000930 | 0.63 |
|  | 9 | PVX_097680 | 0.63 |

Abbreviations**:** AUC = Area under the ROC curve.
