# Supplementary material for: Heterogeneity in response to serological exposure markers of recent Plasmodium vivax infections in contrasting epidemiological contexts
Source: PLoS Negl Trop Dis. 2021 Feb 16;15(2):e0009165. doi: 10.1371/journal.pntd.0009165 (PMC7909627; doi:10.1371/journal.pntd.0009165)
Supplement: S6 Table — (DOCX) [file pntd.0009165.s015.docx]

| **Table S6. Geometric mean titer of 34 SEM across the study sites.** | | | | | | | | | | | | |
| --- | --- | --- | --- | --- | --- | --- | --- | --- | --- | --- | --- | --- |
| **Antibody response** | **Negative controls** | | | **Thailand** | | | **Brazil** | | | **Peru** | | |
|  | **GMT*** | **95% CI*** | | **GMT** | **95% CI** | | **GMT** | **95% CI** | | **GMT** | **95% CI** | |
| PVX_099980 | 0.33 | 0.04 | 17.19 | 0.59 | 0.04 | 20.00 | 1.80 | 0.06 | 20.00 | 4.02 | 0.14 | 20.00 |
| PVX_096995 | 0.16 | 0.02 | 8.35 | 0.19 | 0.02 | 5.50 | 0.29 | 0.03 | 7.59 | 0.89 | 0.04 | 20.00 |
| PVX_101530 | 0.49 | 0.08 | 20.00 | 1.62 | 0.24 | 20.00 | 2.31 | 0.21 | 20.00 | 6.35 | 0.85 | 20.00 |
| PVX_097715 | 0.88 | 0.13 | 20.00 | 0.38 | 0.06 | 5.27 | 0.24 | 0.04 | 4.49 | 0.39 | 0.06 | 7.76 |
| PVX_094830 | 0.21 | 0.04 | 9.38 | 1.08 | 0.16 | 20.00 | 1.30 | 0.13 | 20.00 | 2.96 | 0.28 | 20.00 |
| PVX_112670 | 0.81 | 0.12 | 20.00 | 0.50 | 0.09 | 8.13 | 0.70 | 0.11 | 20.00 | 1.20 | 0.18 | 20.00 |
| PVX_090970 | 1.17 | 0.14 | 20.00 | 1.83 | 0.39 | 20.00 | 1.88 | 0.24 | 20.00 | 1.40 | 0.21 | 19.78 |
| PVX_084720 | 0.39 | 0.09 | 12.68 | 0.49 | 0.11 | 5.89 | 0.70 | 0.14 | 12.39 | 2.40 | 0.52 | 20.00 |
| PVX_003770 | 0.21 | 0.03 | 7.95 | 0.43 | 0.04 | 10.33 | 0.36 | 0.03 | 12.55 | 0.62 | 0.07 | 20.00 |
| PVX_092990 | 1.91 | 0.30 | 20.00 | 2.57 | 0.53 | 20.00 | 3.21 | 0.59 | 20.00 | 2.95 | 0.52 | 20.00 |
| PVX_091710 | 0.70 | 0.14 | 20.00 | 0.86 | 0.20 | 10.28 | 1.25 | 0.25 | 20.00 | 0.63 | 0.10 | 14.30 |
| PVX_087885 | 0.23 | 0.04 | 9.77 | 0.40 | 0.06 | 8.81 | 0.56 | 0.08 | 12.98 | 1.77 | 0.26 | 20.00 |
| PVX_082700 | 0.07 | 0.02 | 1.25 | 0.34 | 0.03 | 20.00 | 0.15 | 0.02 | 7.58 | 0.77 | 0.04 | 20.00 |
| PVX_082650 | 0.09 | 0.02 | 6.30 | 0.80 | 0.08 | 15.12 | 1.66 | 0.12 | 20.00 | 3.00 | 0.10 | 20.00 |
| PVX_094255 | 0.32 | 0.04 | 20.00 | 2.89 | 0.59 | 20.00 | 3.39 | 0.69 | 20.00 | 5.10 | 1.11 | 20.00 |
| PVX_097680 | 0.22 | 0.02 | 8.88 | 0.19 | 0.03 | 3.45 | 0.20 | 0.03 | 3.24 | 0.38 | 0.06 | 10.20 |
| PVX_097625 | 0.12 | 0.02 | 3.98 | 0.19 | 0.02 | 3.84 | 0.07 | 0.02 | 1.80 | 0.19 | 0.02 | 7.91 |
| PVX_082670 | 0.07 | 0.02 | 1.57 | 0.16 | 0.03 | 2.54 | 0.25 | 0.03 | 6.38 | 0.27 | 0.02 | 12.50 |
| PVX_082735 | 0.13 | 0.02 | 2.02 | 0.15 | 0.02 | 5.09 | 0.12 | 0.02 | 3.60 | 0.39 | 0.03 | 20.00 |
| PVX_121897 | 2.56 | 0.39 | 20.00 | 0.32 | 0.04 | 6.05 | 0.24 | 0.03 | 7.92 | 0.59 | 0.06 | 12.91 |
| PVX_090330 | 0.23 | 0.04 | 9.40 | 0.29 | 0.06 | 3.37 | 0.22 | 0.03 | 4.87 | 0.36 | 0.07 | 5.75 |
| PVX_123685 | 0.28 | 0.04 | 9.04 | 0.33 | 0.06 | 4.17 | 0.36 | 0.05 | 5.96 | 0.88 | 0.11 | 17.22 |
| PVX_097720 | 0.10 | 0.02 | 2.66 | 0.14 | 0.02 | 2.89 | 0.17 | 0.02 | 3.22 | 0.38 | 0.02 | 11.58 |
| PVX_000930 | 0.08 | 0.02 | 3.68 | 0.15 | 0.02 | 4.44 | 0.20 | 0.02 | 7.20 | 0.50 | 0.03 | 18.08 |
| PVX_090240 | 1.02 | 0.20 | 20.00 | 2.12 | 0.49 | 20.00 | 1.38 | 0.09 | 20.00 | 1.30 | 0.06 | 20.00 |
| PVX_110810A | 0.12 | 0.02 | 10.19 | 0.17 | 0.02 | 7.78 | 0.44 | 0.03 | 20.00 | 0.98 | 0.04 | 20.00 |
| AAY34130.1 | 0.06 | 0.02 | 4.42 | 0.13 | 0.02 | 4.16 | 0.28 | 0.02 | 20.00 | 0.70 | 0.04 | 20.00 |
| KMZ83376.1 | 0.11 | 0.02 | 7.47 | 0.44 | 0.02 | 17.25 | 0.29 | 0.02 | 20.00 | 2.17 | 0.22 | 20.00 |
| PVX_095055 | 0.16 | 0.02 | 12.30 | 0.27 | 0.02 | 15.81 | 0.42 | 0.03 | 13.40 | 0.99 | 0.05 | 20.00 |
| PVX_098585 | 0.62 | 0.08 | 17.12 | 0.60 | 0.08 | 14.58 | 0.80 | 0.09 | 20.00 | 1.74 | 0.21 | 20.00 |
| PVX_121920 | 0.49 | 0.09 | 20.00 | 0.53 | 0.09 | 18.89 | 1.12 | 0.16 | 20.00 | 3.64 | 0.46 | 20.00 |
| PVX_094255B | 0.08 | 0.02 | 6.65 | 0.28 | 0.02 | 12.24 | 0.40 | 0.02 | 18.75 | 1.96 | 0.06 | 20.00 |
| PVX_087885A | 0.07 | 0.02 | 1.76 | 0.59 | 0.07 | 5.96 | 2.15 | 0.36 | 20.00 | 0.95 | 0.11 | 17.62 |
| PVX_092995 | 0.33 | 0.04 | 10.12 | 0.18 | 0.02 | 4.15 | 1.51 | 0.22 | 20.00 | 0.42 | 0.03 | 9.20 |
| Abbreviations: GMT = Geometric mean titer; 95% CI = 95% confidence interval. *Values are in relative antibody units (RAU) interpolated from standard curves using a 5PL logistic regression model. RAU were values going from 0.02 to 0.00000195. RAU values were multiplied by 1000 to ease reading. | | | | | | | | | | | | |
|  |  |  |  |  |  |  |  |  |  |  |  |  |
|  |  |  |  |  |  |  |  |  |  |  |  |  |
