# Supplementary material for: Heterogeneity in response to serological exposure markers of recent Plasmodium vivax infections in contrasting epidemiological contexts
Source: PLoS Negl Trop Dis. 2021 Feb 16;15(2):e0009165. doi: 10.1371/journal.pntd.0009165 (PMC7909627; doi:10.1371/journal.pntd.0009165)
Supplement: S7 Table — (DOCX) [file pntd.0009165.s016.docx]

| **Table S7. Correlation between antibody titers and age.** | | | | | | |
| --- | --- | --- | --- | --- | --- | --- |
| **Antibody response** | **Thailand** | | **Brazil** | | **Peru** | |
|  | **Spearman's rho** | ***p* value** | **Spearman's rho** | ***p* value** | **Spearman's rho** | ***p* value** |
| PVX_099980 | 0.249 | **** | 0.343 | **** | 0.218 | **** |
| PVX_096995 | 0.231 | **** | 0.343 | **** | 0.428 | **** |
| PVX_101530 | 0.113 | ** | 0.126 | *** | 0.285 | **** |
| PVX_097715 | 0.218 | **** | 0.315 | **** | 0.290 | **** |
| PVX_094830 | 0.424 | **** | 0.152 | **** | 0.306 | **** |
| PVX_112670 | 0.297 | **** | 0.228 | **** | 0.283 | **** |
| PVX_090970 | 0.050 | n.s | 0.190 | **** | 0.229 | **** |
| PVX_084720 | 0.124 | *** | 0.104 | ** | 0.228 | **** |
| PVX_003770 | 0.462 | **** | 0.273 | **** | 0.422 | **** |
| PVX_092990 | 0.013 | n.s | 0.146 | **** | 0.210 | **** |
| PVX_091710 | 0.128 | *** | 0.202 | **** | 0.229 | **** |
| PVX_087885 | 0.236 | **** | 0.240 | **** | 0.248 | **** |
| PVX_082700 | 0.386 | **** | 0.300 | **** | 0.331 | **** |
| PVX_082650 | 0.494 | **** | 0.118 | *** | 0.482 | **** |
| PVX_094255 | 0.361 | **** | 0.301 | **** | 0.380 | **** |
| PVX_097680 | 0.124 | *** | 0.284 | **** | 0.347 | **** |
| PVX_097625 | 0.311 | **** | 0.302 | **** | 0.228 | **** |
| PVX_082670 | 0.477 | **** | 0.310 | **** | 0.391 | **** |
| PVX_082735 | 0.342 | **** | 0.371 | **** | 0.336 | **** |
| PVX_121897 | -0.103 | ** | 0.019 | n.s | 0.143 | *** |
| PVX_090330 | 0.124 | *** | 0.263 | **** | 0.330 | **** |
| PVX_123685 | 0.240 | **** | 0.235 | **** | 0.203 | **** |
| PVX_097720 | 0.363 | **** | 0.285 | **** | 0.408 | **** |
| PVX_000930 | 0.356 | **** | 0.347 | **** | 0.281 | **** |
| PVX_092995 | 0.292 | **** | 0.132 | **** | 0.200 | **** |
| PVX_087885A | 0.390 | **** | 0.173 | **** | 0.293 | **** |
| PVX_121920 | 0.202 | **** | 0.333 | **** | 0.362 | **** |
| PVX_094255B | 0.590 | **** | 0.368 | **** | 0.436 | **** |
| PVX_095055 | 0.350 | **** | 0.319 | **** | 0.270 | **** |
| PVX_090240 | 0.479 | **** | 0.003 | n.s | 0.498 | **** |
| AAY34130.1 | 0.296 | **** | 0.391 | **** | 0.314 | **** |
| KMZ83376.1 | 0.615 | **** | 0.359 | **** | 0.466 | **** |
| PVX_098585 | 0.292 | **** | 0.353 | **** | 0.397 | **** |
| PVX_110810A | 0.248 | **** | 0.381 | **** | 0.300 | **** |
| Abbreviations: n.s : not significant, **: p <0.01, *** : p <0.001, **** : p <0.0001 | | | | | | |
