# Supplementary material for: Heterogeneity in response to serological exposure markers of recent Plasmodium vivax infections in contrasting epidemiological contexts
Source: PLoS Negl Trop Dis. 2021 Feb 16;15(2):e0009165. doi: 10.1371/journal.pntd.0009165 (PMC7909627; doi:10.1371/journal.pntd.0009165)
Supplement: S9 Table — (DOCX) [file pntd.0009165.s018.docx]

| **Table S9. Overall antibody response and number of detected blood- stage infections.** | | | | | | | | | |  |  |  |  |
| --- | --- | --- | --- | --- | --- | --- | --- | --- | --- | --- | --- | --- | --- |
|  | **No infection** | | | **1 infection** | | | **2 infections** | | | **> 2 infections** | | |  |
| **Site** | **GMT*** | **95% CI*** | | **GMT** | **95% range** | | **GMT** | **95% CI** | | **GMT** | **95% CI** | | ***p* value** |
| Negative controls | 0.25 | 0.02 | 15.89 |  |  |  |  |  |  |  |  |  |  |
| Thailand | 0.38 | 0.03 | 11.64 | 0.71 | 0.04 | 19.46 | 0.96 | 0.06 | 20.00 | 0.38 | 0.03 | 11.64 | <0.001 |
| Brazil | 0.45 | 0.02 | 20.00 | 0.86 | 0.03 | 20.00 | 0.99 | 0.04 | 20.00 | 1.09 | 0.05 | 20.00 | <0.001 |
| Peru | 0.69 | 0.04 | 20.00 | 1.30 | 0.07 | 20.00 | 1.24 | 0.05 | 20.00 | 1.98 | 0.09 | 20.00 | <0.001 |
| Abbreviations: GMT = Geometric mean titer; 95% CI = 95% confidence interval. * Data of relative antibody titers interpolated from standard curves were geometric mean with 95% confidence interval (CI). IgG levels were multiplied by 1000. Participants were categorized according to their number of qPCR detection in the last year. Kruskal-Wallis test by ranks was used for analysing the variance among groups. | | | | | | | | | | | | | |
