# Supplementary material for: Heterogeneity in response to serological exposure markers of recent Plasmodium vivax infections in contrasting epidemiological contexts
Source: PLoS Negl Trop Dis. 2021 Feb 16;15(2):e0009165. doi: 10.1371/journal.pntd.0009165 (PMC7909627; doi:10.1371/journal.pntd.0009165)
Supplement: Attachment — Submitted filename: Response_Pv_SEMs_contrasting_epi_settings_311220.docx [file pntd.0009165.s019.docx]

**Malaria: Parasites and Hosts Unit**

Institut Pasteur

25-28 Rue du Dr Roux

Paris 75015

France

**MSc Jason Rosado**

javier.rosado@pasteur.fr

30^th^ December 2020

Dear Editor,

We would like to resubmit a revised version of our manuscript – “Heterogeneity in response to serological exposure markers of recent *Plasmodium vivax* infections in contrasting epidemiological contexts” – for consideration for publication in PLOS Neglected Tropical Diseases.

We would like to thank the editors and reviewers for their time spent considering the manuscript and their helpful insights. All of the reviewers’ comments have been addressed on a point-by-point basis below. We believe that the changes made in response to these suggestions have led to a substantially improved manuscript.

We look forward to your response,

Yours sincerely,

MSc Jason Rosado, on behalf of the authors

**EDITORIAL COMMENTS**

1. We noticed that you used “data not shown" in the manuscript, we do not allow these references. The PLOS data access policy requires that all data be either published with the manuscript or made available in a publicly accessible database. Please amend the supplementary material to include the referenced data or remove the references. Please make a note of this change in your response to reviewers.

We have added the references regarding the validation of the Luminex protocol (line 191).

2. We do not publish any copyright or trademark symbols that usually accompany proprietary names, eg (R), (C), or TM (e.g. next to drug or reagent names). Therefore please remove all instances of trademark/copyright symbols throughout the text, including 'E.Z.N.A. ®' 'PerfeCTa® SYBR®' 'Milliplex®' and 'Luminex®' on pages 15 - 16.

We have removed all trademark symbols in the text.

3. Please remove the figures embedded in your manuscript file leaving only the separate figures files. If you are using a LaTeX source file, please remove \includegraphics to remove the figures from your PDF.

We have removed the figures from the main manuscript and are presented as separate figures now.

**REVIEWERS' COMMENTS**

**Reviewer #1:** Minor comments

-	Line 87- what about RDT? As you are advocating a serological rapid test, would be good to compare it to the equivalent rapid test that is currently available.

Our team’s main aim for now is understanding the epidemiology behind the antibody response signature and use that knowledge to understand the strengths and limitation and improve the signature. There are currently no rapid tests that can detect recent exposure and thus hypnozoite carriage. Our team is developing a sero-RDT and a future aim will be to deploy and evaluated the SeroTAT tool in the field.

-	Line 135- how were the volunteers chosen? Randomly? What was the loss to follow up- was that the reason for the extra participants in the third year?

We have edited the paragraph regarding the selection of enrolled participants (lines 134-155).

-	When stating the numbers of active case detection visits- presumably this was maximum? Not everyone would have been available for all.

Indeed, 37 active case detection visits were the maximum number of visits in the Peruvian cohort. The loss of follow-up in this cohort was described by Rosas-Aguirre *et al*, 2020. This information is provided in lines 140 -155.

-	State a bit more information re. the Thai and Brazilian cohorts- all ages, randomly selected? Why weren’t the full cohorts from Longley et al included?

We have updated the section regarding the cohorts’ description (lines 166-171). Since our study aimed to compare the antibody responses in the Peruvian cohort to cohorts with similar designs, we did not include the Solomon Islands as it enrolled pediatric participants only.

-	Could you provide a justification for the use of the linear model? As it is a cohort I would expect to see a model accounting for time under observation- but I think you have reduced your explanatory variables to binary? At some point you report odds ratios suggesting you have used a cut off (not defined) to designate responses as negative/positive- would this be a more appropriate way to look at risk factors (though I acknowledge you lose information by reducing responses to binary)? I also feel that including age as a continuous variable in the model may be inappropriate- I think a categorical variable would more appropriately capture the dynamic changes over age group.

Linear models were applied to log-transformed antibody data, an appropriate assumption given the continuous log-Normal distribution of antibody data. On the other hand, dichotomised antibody data simplifies analysis with the cost of losing information. We do not use a model accounting for the time under observation because we selected participants with full follow-up for 12 months. In our linear model, we selected age as a continuous variable because of the approximately linear relationship between antibody levels and the log of age. While age categories would facilitate interpretation of this association, the selection of age categories are still subjective, and there is no correct way to select them.

-	Line 184- it is not clear to me what this analysis entails- by taking into account, do you mean adding in to the model?

The aim of this analysis was to understand what epidemiological factors could explain the antibody levels in the Peruvian cohort. Our analysis was retrospective because it used qPCR data of the 13 months preceding the last time-point (reference point). The model was corrected by age, community, gender, clinical infections in the last 6 months, education level, time living in the community and working as a farmer (Table II in S1 file). We edited this section (lines 208 -212).

-	Line 185: D0 is a bit confusing as it’s actually at the end of the cohort- consider renaming.

We agree that the term DO is confusing; we renamed it as the last time-point (line 211).

-	Line 189- couldn’t Peru have been longer because of the three year cohort? It does seem further data is used in your figures?

We used three years' data follow up in the figures; however, we focused our analysis in the last year of follow-up to compare to the other two cohorts, which both had only 12 months of follow-up.

-	Line 188: Is there a reason 9 months was picked as the cut off for this?

Nine months was used because this cut-off takes into account that all P. vivax strain (with the possible exception of the hybernans strain now restricted to Korea) will experience their first relapse within no more than 9 months following the primary mosquito-bite derived infection. Thus, a 9 months recent exposure period allows to capture almost all people carrying hyponzoites and thus at risk of experiencing a relapse. This paragraph was added in methods section (lines 217-220).

-	Could you give a fuller explanation of disjoint training (line 196)?

We have amended this section. In this work we did not cross validate. The algorithm was described by Longley and White MT *et al.*2020 (line 226) [1].

-	Is this a cut off determined from your negative samples or from the models? How was the cut off value determined?

Rather than selecting a single cut off value, we varied the cut off across the range of measured antibody levels. These cutoff values were compared against both the negative and positive samples to generate ROC curves.

-	Line 199- clarify whether age was used as a linear var- it seems in the supplementary that it was also log?

Age was log_10_ transformed in both the lineal model of variance in the main manuscript, and the multivariate regression model in the supplementary. We are clarifying this paragraph to the method section (line 232).

**Reviewer #2:**

Throughout the objectives, objectives are clear, well-defined, and analyzed appropriately.

All populations are well-described in text, or in refs (prior cohorts), and all ethical issues are covered.

** Edits required:

Line 134. The description of both "home-to-home" and "community-based" screening should expanded, to make the sampling frames and enrollment processes clear.

We have edited the paragraph regarding the selection of enrolled participants (line 136).

Line 135. While recent anti-malaria drug use was an exclusion criterion, what the standard-of-care during this cohort, and what exclusions if any existed for antimalarials during the follow-up period? (Table 1).

After checking the protocol of the cohort study, we are removing the sentence about the recent use of anti-malaria drug (line 134). Participants excluded from the cohort had the following characteristics: being younger than three years old and not giving informed consent to use a blood sample. Information about treatment is provided in the manuscript (lines 147-151). We apologize for this oversight.

Line 187. Was sensitivity analysis performed for the choice of 9-months as the cutpoint for "recent exposure" ?

See comment above. This information is provided in methods section (lines 217-220). We also included the analysis of diagnostic performance across different timeframes in the supplementary information (Fig VIII in S1 File).

Line 195. References should be added for LDA (in general), and the justification for the 1/3+2/3 split and 200 cross-validations.

We have amended this section. In this work we did not cross validate. The algorithm was described by Longley and White MT *et al.*2020 (line 226) [1].

An overview map of the study region would be welcomed, but is not absolutely essential.

This map has been provided by Rosas-Aguirre *et al.* 2020, which is cited as part of the study description.

** Minor edits:

Line 128: "Malaria" need not be capitalized.

We have edited that term.

--------------------

**Results**

**Reviewer #1:**

-	Line 214: ratio, not rate.

We have edited that term (line 247).

-	Line 215: if short duration in a community is a risk factor- does this not suggest that they were infected elsewhere? This may limit the usefulness of including them in a population study looking at transmission in a given setting- could be something to bring up in the discussion re the importance of understanding the population context.

Migration affected the cohort's design with loss of follow up and entry of new residents (who were not included in the study). Migration may have affected the incidence and prevalence either by introducing new strains or by the introgression of malaria-naïve individuals [2]. Unlike Lupuna, Cahuide is vulnerable to imported malaria due to work related-human mobility and social interactions within and between other endemic communities [3]; however, the transmission dynamics in the Peruvian amazon goes beyond our work aims. We have now mentioned this as a limitation of our study (lines 480-485).

-	Line 224- given that these are samples repeated on individuals, who presumably were not treated (?), could you state how many individuals were infected at any point during the cohort, rather than just the number of positive samples.

See table 1; we have added the information about the number of participants infected in the last 9 months (line 258).

-	Table 1: time since previous infection numbers are confusing- is this at the end of the follow up?

Time since the previous infection is the time elapsed between the last blood-stage detection and the end of follow up (serum sample collection).

-	Why are you presenting statistical differences between the two areas sampled- do you then include this in the model? Or is it to highlight a higher and lower transmission place? Maybe make this clearer in the text- you subsequently put all the Peru data together but maybe it could be kept separate considering the differences in transmission intensity between the two settings (maybe too few samples?)

The reviewer is correct that the Peruvian samples were pooled in the analysis because of samples numbers in order to optimize statistical power. Nonetheless, we felt it was instructive to also report stratified results.

-	It seems odd to me that you’re presenting prevalence throughout rather than incidence- are you considering each monthly visit as a cross-sectional survey, rather than a cohort? Is there any instances in your analysis where you should be account for the fact that you have multiple infections from the same person? And also refer to previous comment re. whether you know if these are multiple infections or the same infection detected on subsequent visits.

We have not presented incidence data in this article because we were unable to differentiate between new incident infections, relapses and continuations of previous infections. No genotyping data were available by the time we executed the study. In our multivariate regression analysis, we accounted for the number of positive qPCR detections in the last year of follow up as a surrogate measure of incidence of infection.

-	Line 241- why do you pick the age of 40 here?

In our analysis, people older than 40 years have higher antibody levels than younger people (<40 years old).

-	When you are talking about infected in the previous x days- make clear that this is at the end of the cohort (if I have understood correctly)- again, D0 makes this a bit confusing- which you’d think would be at the beginning, not the end.

We have edited the caption in Fig 1.

-	If you are using an odds ratio- then you must have a binary variable for your antibody responses- but you have not mentioned determining a cut off anywhere? I couldn’t find reference for this logistic model in your methods.

The odds ratio is produced via the logistic regression model. Notably, the outcome is binary (i.e. currently infected), and the predictor variables can be either continuous or categorical. The information about variables included in the model are mentioned now in the legend of Table I in S1 File. For antibody responses, the odds ratio refers to the changes in odds of the binary outcome for each unit change of the predictor variable (e.g. log_10_ antibody response).

-	Fig 2- your age brackets are misaligned.

We have edited it.

-	Relative antibody units are all negative- is this expected?

We normalized the Median Fluorescence Intensity (MFI) data by extrapolating the samples’ MFI to a standard curve that went from 1/50 (0.02) to 1/512 000 (0.00000195) dilutions. The extrapolated dilutions or relative antibody units (RAU) are continuous data that goes from 0.02 to 0.00000195. The RAU data is log_10_ transformed for further analysis. The log_10_ RAU are thus all negative values. We have edited the labels of RAU values in the Fig2 to ease interpretation by readers.

-	Line 251: are these results from models with just the one explanatory factor? I think age should be controlled for a priori as you are probably looking at previous exposure- which is potentially not what you are interested in here- do the SEMs get less specific with age group?

As shown in table II in S1 file, our interest was to know what factors other than multiple infections could have influenced the antibody responses. The epidemiological factors included in the multivariate model were the following: age, gender, time living in community, community, number of clinical episodes. We found that antibody levels to some SEMs increased with age and could reflect either the relationship to age risk factors such as job exposure or cumulative exposure in the lifetime.

-	In para staring line 251 you talk about previous 6 months- but previously it has been 9 months- why the discrepancy? Please justify the 9 month cut off.

In the multivariate model (Table II in File S1), we preferred to count the number of blood-stage detections in both the previous six months prior to the end of follow up, and in the first six months of follow up as it was a better metric to evaluate the intensity of exposure in 1 year follow up. We have added this information in methods (lines 207-209). The justification for 9 months cut-off is provided in methods section (lines 216-219).

-	Line 271: what does positively correlated to time since previous infection mean- that would suggest to me that the longer ago the infection, the higher the antibody responses- perhaps needs rewording?

We meant that individuals with more recent infections had higher antibody levels than individuals with older infections. We edited that phrase (lines 307-308).

-	Line 272: what are blood stage detections? PCR +ve?

Yes, blood stage detections are positive qPCR results.

-	Figure 6: I think your Y axis need to highlight that they’re looking at the association with those things- not actually those things- at first I was surprised that Peru seemed to have lower mean titres than the other settings.

We mentioned in the caption that this analysis is looking at the association between cumulative PCR prevalence and antibody titers, performance and variance. Peru had the highest antibody titers, the units are geometric mean titer of log_10_ RAU. We have edited figure 6 as suggested.

-	Figure 6- these dots are very large- is that representative of any uncertainty? Or are they just large?

The dots size does not represent any uncertainty. The size was chosen to ease visualization.

**Reviewer #2:** ** Edits required:

Line 236. As the geo mean responses overlapped, the villages were combined for analysis; however, the residence time in the villages differs sharply. Was this covariate added in the multivariable models?

In exploratory analysis, we found positive correlation between the years living in the community and the antibody levels for some antigens. We added the covariate time in community (continuous) in the multivariable model (Table II in S1 File). Unlike Lupuna, Cahuide is vulnerable to imported malaria due to work related-human mobility and social interactions within and between other endemic communities (Rosas-Aguirre, 2020); however, the transmission dynamics in the Peruvian amazon goes beyond our work aims.

Line 244 (Figure 2). The metric "immunogenicity" (y-axis) on should be clarified- is it quantitative or just a general sorting?

Immunogenicity of SEMs was defined as the geometric mean of RAU across all participants in the Peruvian cohort. We used this quantitative metric to sort the antibody responses in the Peruvian cohort. A brief explanation is provided in the caption of Figure 2 (lines 277-279).

Line 323ff. Are the 95% CIs for the "percent explained variation" estimates overlapping here? That is, are these differences meaningful?

The “percent explained variation” was calculated using the calc.relimp R package to estimate the proportion of variation in antibody responses attributable to the considered factors. These estimates do not include uncertainty.

Line 339ff. While the authors do state the limitations and caveats of these 3-data point comparisons, these comparisons should be further noted to be "exploratory." The relapse rates, EIRs, and recent transmission regimes are so radically different in these settings make comparisons difficult.

We agree. We have edited the paragraph (line 387)

--------------------

**Conclusions**

**Reviewer #1:**

-	Line 381: you state that both memory and antibody cells appear to be long-lived- do you have a reference for this? You are measuring antibody cells- not memory cells- so could it not be that a higher response is developed in the higher transmission setting and therefore it may be waning but still appears high after a longer period post-infection- this is described in the following sentences in your discussion. I’m not sure you know whether memory cells are responsible.

Memory, plasma cells and antibody levels have not been rigorously investigated in high transmission settings. We added a reference (lines 422-423). We did not measure antibody cells or memory cells, but antibody responses; however, we referred that the antibody longevity is maintained by memory B cells that rapidly proliferate and differentiate into antibody secretory cells upon re-exposure. We think that new infections and hypnozoite relapses could boost immunological memory, therefore antibody responses.

-	Line 401: I think this sentence needs rewording- if you didn’t test PvMSP10 then you can’t confirm those results- you could say that you see similar patterns with other MSP markers?

We did not test PvMSP10; however, we saw similar patterns in terms of diagnostic performance and age influence with other MSP markers (PvMSP7 and PvMSP3). We have edited this paragraph (lines 441-444)

-	Line 413- if antibodies have a mean half life of 4 months, won’t you potentially be missing these if you at people who had an infection 9 months ago? I’m still unclear on the justification of this time lapse.

Four months was the estimated antibody half-life of anti-PvRBP2b in participants from low transmission settings who were treated with primaquine after diagnosis, thus clearing liver-stage parasites and without possibility to relapse. A four month half-life will lead to a 50% reduction in antibody levels after four months, a 75% reduction after eight months and so on. Importantly, antibody levels will still be measurable for a substantial period of time.

-	Legend for time since previous infection- could you reverse your legend so that <1 month is at the other end to reflect the bar placement?

We have edited it.

-	Table 1: supplementary- are these logistic regressions controlled for any other variables?

Logistic regression models were adjusted by the following covariates: log_10_ (age), gender and working as farmer. We are adding this information for better understanding in Table 1 in S1 File.

-	Table II: supplementary- Did you look at each of these risk factors separately? Or just put everything in the model at once?

We put all these risk factors in the model at once. Only the covariates that remained significant are indicated by asterisks.

-	Table VI: It wasn’t clear to me why the negative controls were multiplied by 1000 and isn’t mentioned in methods.

As explained lines above the RAU were values going from 0.02 to 0.00000195. To ease the reading of these values we multiplied by 1000. We are adding this information in the caption of Table VI in S1 File.

**Reviewer #2:** The conclusions largely supported, with one exception.

** Edits required.

Discussion on the geographic variation in parasite populations should be expanded, as this has direct implications for assessing how much of the observed variation is due to transmission intensity as proposed, and how much may be due to immunogenicity of parasite sub-populations. This study cannot and was never intended to address this issue, but there should be greater consideration of this potential confounding.

<https://www.ncbi.nlm.nih.gov/pmc/articles/PMC5552344/>

<https://www.ncbi.nlm.nih.gov/pmc/articles/PMC3713979/>

<https://www.ncbi.nlm.nih.gov/pmc/articles/PMC5347536/>

<https://www.ncbi.nlm.nih.gov/pmc/articles/PMC4021508/>

<https://www.ncbi.nlm.nih.gov/pmc/articles/PMC5809496/>

We have added a paragraph on the limitation of our study on the parasite genetic diversity (lines 474-478).

--------------------

**Editorial and Data Presentation Modifications?**

Use this section for editorial suggestions as well as relatively minor modifications of existing data that would enhance clarity. If the only modifications needed are minor and/or editorial, you may wish to recommend “Minor Revision” or “Accept”.

Reviewer #1: (No Response)

Reviewer #2: (No Response)

**Summary and General Comments**

**Reviewer #1:** This paper summarises a serological dataset from Peru and evaluates responses to a panel of P. vivax antigens- assessing correlation with age, number of infections and time since last infection. The paper aims to validate the use of specific antigens, or combinations of antigens, to be used as markers of recent exposure to P. vivax. The work mainly focusses on a cohort in Peru- but brings in previously analysed datasets from different transmission intensity settings in Thailand and Brazil. The paper is well written and interesting though there is a lot of information to summarise and this isn’t always achieved in the clearest way. I enjoyed the paper and think the data is important and the analysis justified. However, I have a few major comments which need addressing and quite a lot of minor comments which I think would make things clearer for the reader.

**Major comments**

-	The number of detectable infections doesn’t seem like a correct measure to me. As far as I understand it, the people in the cohorts were not treated as these infection measures were done by PCR (so likely with some time delay). In which case, what you are measuring might not be multiple infections, but the same chronic infection? It’s not mentioned whether each time they were positive was counted as a new infection or not- please clarify that in the methods. I would also consider removing this as a metric if it is that you cannot be sure whether each positive PCR is actually a new infection or not. Is there any data on treatment in the cohort? Did people oscillate between PCR negative and positive over different visits? It would be interesting to know more about who is most likely to be getting infections.

In our work, we counted every positive qPCR result as a positive blood-stage detection and this give us a metric to evaluate the association between the exposure to *P. vivax* and antibody titers (line 208 -210). In the Peruvian cohort, participants were diagnosed by passive case detection (PCD) and active case detection (ACD). Only participants with microscopically confirmed infections during PCD or ACD were treated with Chloroquine for 3 days (10 mg/g on days 1 and 2, and 5 mg/kg on day 3) plus primaquine for 7 days (0.5 mg/kg per day). Treatment adherence was monitored by trained staff in health posts when in PCD. In contrast to PCD patients, ACD patients self-reported a full treatment adherence [3] (added in lines 147-150).

Given that submicroscopic infections were detected by qPCR only and were not treated, they possibly indicate either continuing infections, new infections or hypnozoite relapses. However, genotyping studies in other endemic settings showed that the duration range of individual *P. vivax* blood-stage infections is 24 - 29 days [4].Based on this evidence, it is thus more likely that monthly blood-stage detections reflects either new infections or relapses rather than chronic infections. However, we do recognise that without genotyping those blood-stage infections, we cannot tell between relapses, recrudescence and re-infection. There were participants whose qPCR results oscillated between positive and negative. The pattern of oscillation is associated with epidemiological characteristics of the studied population; however, the dynamic of infection was not the main goal of our study and has been previously described [3]. Nevertheless, the number of positive PCRs is likely to be closely correlated with the incidence of bloodstage infections.

-	Given that the need for a tool to demonstrate malaria elimination is highlighted several times- I think the seemingly potential positive responses amongst your negative sera should be addressed. This obviously highlights the risk of non-specific responses which is not mentioned in the manuscript. In Table VI (supplementary): For some antigens, the negative controls have a higher mean titre than the samples from endemic countries- would you recommend the cessation of use of these antigens?

The response to some antigens in the healthy donor group could indicate cross-reactivity with pathogens other than *Plasmodium*. The cross-reactivity issues have been previously addressed [1]. Longley *et al*. reported that proteins with the lower background in the healthy donor group are the ones that provide better classification performance. In our final analysis, we only kept the antigens with better performance; however, modifications of the *P. vivax* constructs that reduce this background such as further purification steps, design of smaller protein fragments and different expression proteins could improve classification by SEMs.

-	The Brazil and Thai cohorts have been previously analysed in Longley et al. It feels like some of that analysis has been duplicated in this paper but without the Solomon Islands data- is there a reason that dataset has not been included (maybe the age range?)? I think at times the comparisons to the other cohorts feels a bit shoehorned in.

Brazil and Thai data were included in the study to better understand the antibody responses in Peru by comparison. We did not include Solomon Islands data because it was a pediatric cohort.

-	Age representing cumulative exposure vs risk behaviour- this is always a complicated thing to pull apart. From the paper, it seems that you don’t consider that age is necessarily a risk factor in Peru (you mention peri-domestic transmission)- in which case would it be useful to look at the ROC for different age categories in Peru. A seroTAT might not be useful looking in all age groups (if adults have been exposed multiple times and therefore have antibodies remaining from an infection of several years ago) but testing children in a household might be a more sens/spec way to identify households with ongoing transmission- alternatively the two settings in Peru might allow you to look at this. I recognise you have incorporated a large amount of variables into your models- but might it be more useful to stratify by age/location, rather than just include everything at the same time. It could be useful for operational guidance too- i.e. test children aged under XX- at the moment having stats for a doubling of age giving a 2.18 increase in antibody levels isn’t too useful (line 253).

We have performed a ROC curve analysis stratified by two age categories: 0-15 and 15+ years old (Fig IX in S1 File). The ROC curves in Peru were further analyzed accordingly to the two communities. In the cohorts of Thailand and Brazil, the diagnostic performance was slightly higher in the group of 0 – 15 years old (Thailand _0 - 15_ AUC: 0.90, Brazil _0 - 15_ AUC: 0.87) than the group of 15+ years old (Thailand _15+_ AUC: 0.83; Brazil _15+_ AUC: 0.81). In both Cahuide and Lupuna, people older than 15 years old (Cahuide: _15+_ AUC: 0.71, Lupuna: _15+_ AUC: 0.66) were better classified than younger people (Cahuide: _0 - 15_ AUC: 0.51; Lupuna: _0 - 15_ AUC: 0.62). The high cumulative exposure in both Peruvian communities prevented a good classification of exposure in the last 9 months even in the younger group. A similar pattern was reported by Rosas-Aguirre *et al.* when explored the diagnostic performance for recent exposure given by PvMSP10 in the same population [5]. The diagnostic performance was better in the group of 15+ years old (_15+_ AUC: 0.72) than the group of 0 – 15 years old (_0 - 15_ AUC: 0.66) [5].This information is now provided in lines 352-357.

**Reviewer #2:**

Overall, this is a extremely well-structured and rigorous set of studies. The only limitations are related to clarity and overall length.

The manuscript overall is quite lengthy, and edits should be considered to maximize the impact.

**References**

1.	Longley RJ, White MT, Takashima E, Brewster J, Morita M, Harbers M, et al. Development and validation of serological markers for detecting recent Plasmodium vivax infection. Nat Med. 2020;26(5):741-9.

2.	Carrasco-Escobar G, Miranda-Alban J, Fernandez-Minope C, Brouwer KC, Torres K, Calderon M, et al. High prevalence of very-low Plasmodium falciparum and Plasmodium vivax parasitaemia carriers in the Peruvian Amazon: insights into local and occupational mobility-related transmission. Malar J. 2017;16(1):415.

3.	Rosas-Aguirre A, Guzman-Guzman M, Chuquiyauri R, Moreno M, Manrique P, Ramirez R, et al. Temporal and micro-spatial heterogeneity in transmission dynamics of co-endemic Plasmodium vivax and Plasmodium falciparum in two rural cohort populations in the Peruvian Amazon. J Infect Dis. 2020.

4.	White MT, Karl S, Koepfli C, Longley RJ, Hofmann NE, Wampfler R, et al. Plasmodium vivax and Plasmodium falciparum infection dynamics: re-infections, recrudescences and relapses. Malar J. 2018;17(1):170.

5.	Rosas-Aguirre A, Patra KP, Calderon M, Torres K, Gamboa D, Arocutipa E, et al. Anti-MSP-10 IgG indicates recent exposure to Plasmodium vivax infection in the Peruvian Amazon. JCI Insight. 2020;5(1).
